# Effect of Temperature and Capsid Tail on the Packing and Ejection of Viral DNA

**DOI:** 10.1371/journal.pone.0052958

**Published:** 2013-01-08

**Authors:** Afaf Al Lawati, Issam Ali, Muataz Al Barwani

**Affiliations:** Department of Physics, College of Science, Sultan Qaboos University, Al Khod, Oman; German Cancer Research Center, Germany

## Abstract

We use a simulation technique based on molecular dynamics and stochastic rotation model to present the effect of temperature and capsid tail on the packaging and ejection processes of semiflexible polymers. We consider two types of solvents, a good solvent, where the polymer is neutral and repulsion interactions among its various sections are favored, and one where the polymer is charged, giving rise to extra electrostatic reaction. For tailless capsids, we find that packing a neutral polymer is slightly slower at higher temperatures whereas its ejection is slightly slower at lower temperatures. We find the same trend for a charged polymer but the effect is noticeably larger. At a high enough temperature, we notice that packing a charged polymer can be stopped. On the other hand, at fixed temperature and regardless whether the polymer is charged, packing is much easier for a capsid with a tail whereas ejection is much slower. The effect of including the tail on the dynamics of a charged polymer, in particular, is rather significant: more packing fraction is facilitated at higher temperatures due to more ordered polymer configuration inside the capsid. In contrast, during ejection the tail traps the last remaining beads for quite some time before allowing full ejection. We interpret these results in terms of entropic and electrostatic forces.

## Introduction

Large forces are required to fully pack nucleic acids inside a viral capsid due to the bending rigidity and electrostatic repulsion between different parts of the molecules. This gives rise to enormous pressures that bacteriophages use to eject their genome to host cells during the early stage of the infection process. The bacteriophage 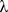 genome, for example, has a persistence length 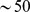 nm [1], and is stored in a capsid of dimensions 50 nm
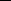
60 nm leading to large internal pressures (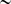 tens of atmospheres).

In the seminal experiment of Smith et. al. [2], the internal capsid force was measured as a function of the amount of the genome in the capsid. It was found that the packing rate is almost constant until 50% of the genome is packed then it reduces to zero at full packing. Also, pauses were observed during packing, due to the motor temporarily loosing its grip on the DNA molecule. They found that when the genome is fully packed, the opposing capsid force reached a maximum of about 50 pN within their experimental conditions.

Other experiments [3] looked at the effect of genome length and ionic state of the buffer. It was found that shorter genomes ejected with lower speeds but shorter total time. On the other hand, the presence of Na^+^ ions in the buffer increased the ejection time. Interestingly, the ejection speed was initially low, where ejection force is highest, becoming a maximum in the intermediate stage of genome ejection, at which point the ejection force is low, leading to the possibility that friction may have an important role in the process (see also Ref. [4], which models three possible mechanisms for the effect of friction).

Some experimental data on T5 phage indicate that lower temperatures possibly result in opening/closing of the head-tail connector and/or to changes in the conformation of the tail leading to appreciable slowdown of ejection [5].

Other experiments also studied the effect of temperature, packaged DNA length and addition of DNA-binding proteins to the host solution in vitro, but on the ejection process of 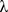 phage DNA using time-resolved static and dynamic light scattering [6]. It was found that the initial ejection rate increases exponentially as a function of temperature. Two possible explanations were advanced to explain this: (a) the tail-receptor proteins adopt a more closed configuration at lower temperatures, or, alternatively,(b) these proteins could close the pore continuously as the temperature is lowered, thereby increasing the friction force on the ejecting DNA. Longer genome lengths and addition of binding proteins also lead to faster ejection rates.

Simulation studies have captured some of the salient features of experiments. The DNA packing at a constant rate into the capsid was simulated using Brownian molecular dynamic simulations [7]. The capsid force opposing DNA packing was found to be small at low packing fractions but increasing significantly when more than 40% of the polymer was packed. It was also found that the DNA gains a spool structure while packing. Other studies have found that the final DNA conformation depends on the shape and the size of the capsid [8]. The packed DNA conformation was also studied by other simulations studies [9]–[11]. Recent molecular-dynamics-based simulations also investigated the effects of various factors on the dynamics of packing and ejection, such as capsid shape [12], solvent quality [13], electrostatic forces [14] and the presence of knots [15].

Forrey and Muthukumar [16] have used Langevin dynamics simulations to look at the structure of packed DNA, packing velocity and the internal energy. Their results suggest that as the polymer is compressed inside the capsid, the above factors become more dependent on the polymer dynamics.

In this paper we report simulation studies on the effect of the temperature and the capsid tail on the packing and ejection processes of neutral and charged, semiflexible polymers from a spherical capsid. We find that temperature plays opposite roles: lower temperatures slow down ejection, as in experiments, but increase packing speed. On the other hand, the capsid tail makes packing easier but ejection harder. These effects are particularly large for a charged polymer.

## Methods

We use molecular dynamics (MD) simulations in which the polymer is coarse grained into 100 beads, each of diameter 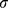, connected by finite extension nonlinear elastic springs (FENE). The FENE potential is
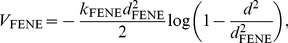
(1)where 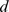 is the distance between two consecutive beads, 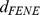 is the maximum extension of the spring and 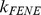 is the strength of the spring. The beads also interact by Lennard-Jones potential in order to generate excluded volume interactions,
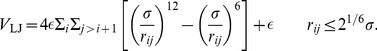
(2)In this potential, 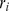 is the position of the 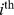 bead and 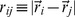. In addition, there is a bending rigidity potential, which incurs a penalty when any section of the polymer tries to bend
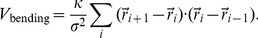
(3)We choose the various parameters in these potentials as 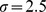 nm, 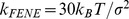 and 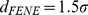. These choices result in a bond length 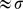. The bending rigidity parameter 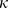 is set so that the persistence length of the semiflexible polymer is about 10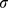 (we note that this is smaller than 20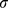, the persistence length of DNA in typical physiological conditions, in order to keep the simulations within reasonable time limits). 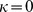 for a flexible polymer. We fix 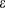 = 1 in simulation units and perform simulations at temperatures 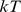 = 1.2, 1.0 and 0.8 (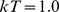 corresponds to 37°C). Finally, the electrostatic potential due to the charge of the DNA backbone is
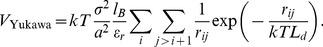
(4)Here, 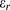 is the solvent relative permeability (80 for water), 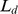 is the Debye screening length and 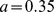 nm. For water at 37°C, 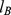, the Bjerrum length, is 0.7 nm and 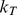 = 4.3 pN nm. We use 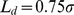 for the charged polymer, corresponding to a screening length of 1.9 nm. For this reason we cut the electrostatic potential at 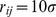, which is much greater than 0.75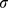. It should be noted that experimentally [17] 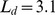 nm corresponds to 100 mM Na^+^. One can achieve smaller screening lengths by adding multivalent ions such as Mg^+2^.

The capsid is modeled as a sphere with a hole that allows the entrance of one bead at a time. The capsid is permeable to the solvent particles, as is the case in experiments [2]. We choose the radius of the sphere to be 3.02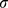. Although this results in a sphere of radius 7.5 nm, which is smaller than viruses found in nature (e.g. the radius of 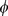
29 is 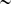
25 nm), it still gives conditions that are relevant to experiments. For example, the packing fraction with this radius is about 0.4, close to natural physiological conditions. It has also been shown [1] that this capsid reproduces the decrease in packing rate, as more of the DNA is filled inside the capsid, and the random pauses observed during packing [2].

In order for the polymer beads not to penetrate the capsid wall, a repulsive force 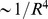 is applied to any bead, whether inside or outside the capsid, whose coordinates are such that 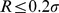, where 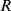 is the radial distance between the bead and the capsid wall.

The viral molecular motor which has to capture the beads and feed them inside the capsid is extremely complex [18]. However, we model it here simply as a constant force at the capsid entrance. The force is directed towards the capsid center and is applied to any bead close to the capsid entrance. We set the force value at 80 simulation units (1 simulation unit = 1.6 pN), corresponding to 128 pN. Such a large force is necessary especially when feeding a charged, semiflexible polymer into the capsid.

**Figure 1:**
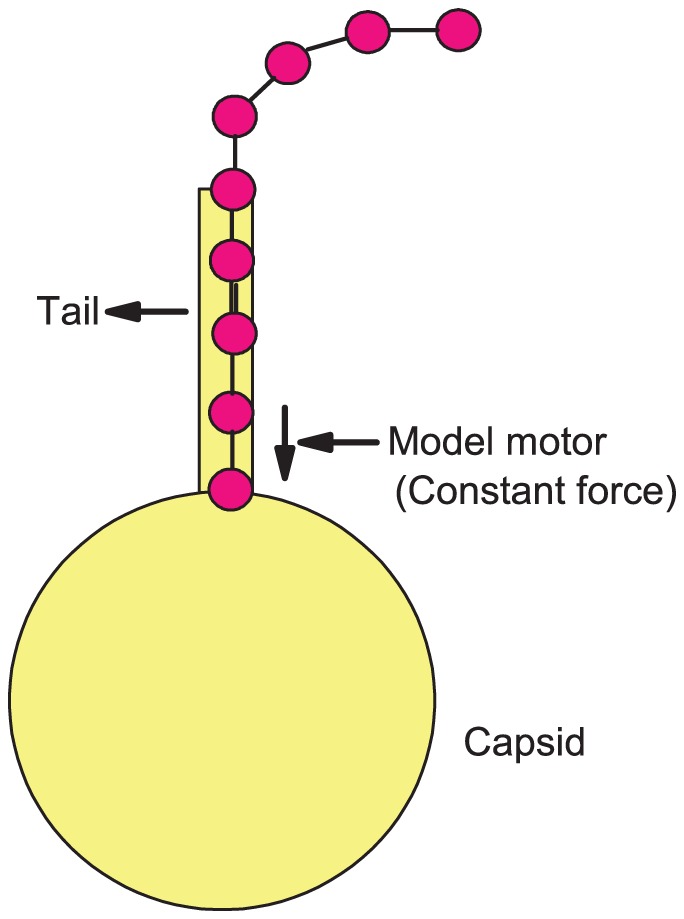
Spherical capsid with model motor.

Phages typically have long tails that are comparable to, or even larger than, the capsid diameter (the 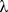 phage, for example, has a capsid diameter of about 60 nm with a tail length approximately 100 nm). We model the phage tail in our simulations as a cylinder of length 6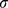 (see Fig. 1), about the diameter of the capsid, and radius 0.7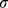. The cylinder is attached to the capsid opening. A bead is retained in the cylinder via a force 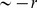, where 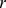 is the radial distance of the bead from the cylinder axis.

The polymer is initially configured so that one bead is inside the capsid and the rest are outside. It is equilibrated in this position for 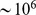 MD time steps before applying the feeding force for packing (1 MD step 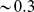 ns for cytosol, which has viscosity 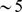 cP at 37°C). After packing, the polymer is equilibrated inside the capsid for 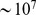 MD steps but with one bead left outside to initiate ejection. Ejection then starts by setting the feeding force to zero.

The solvent particles are modeled using stochastic rotation dynamics [19]. It is a hydrodynamic thermostat that allows momentum transfer between polymer beads due to the interactions between the beads and the solvent particles. This model divides the fluid to point particles that randomly collide, through a collision step, at discrete time steps followed by a streaming step in random directions in continuous space. See Refs. [1], [19] for more details.

## Results and Discussion

### Effect of Temerature

We first consider the effect of temperature on the packing and ejection of neutral (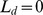) and charged (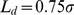) semiflexible polymers from a tailless capsid.

**Figure 2:**
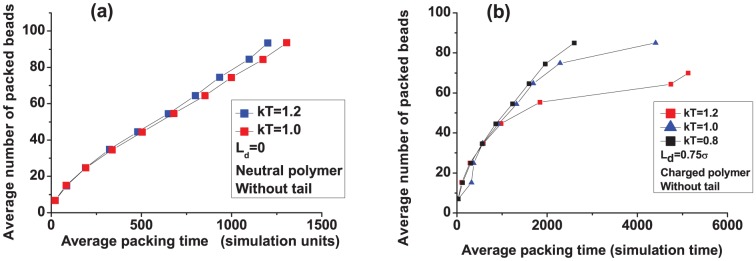
Effect of temperature on the packing of a semiflexible polymer: (a) polymer is neutral and (b) polymer is charged ( 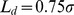
 **).**

**Figure 3:**
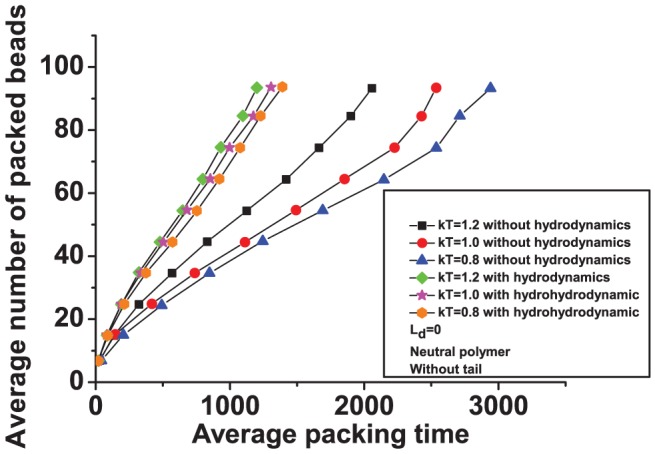
Effect of hydrodynamics on packing of a semiflexible, neutral polymer into a tailless capsid at different temperatures.

In Fig. 2(a) we plot the number of packed beads vs. time at two different temperatures (each curve in this and all subsequent plots is an average over 100 individual polymers, unless stated otherwise; the error bars–standard errors on the mean–are smaller than the symbols in all curves). We find that increasing the temperature from 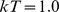 to 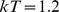 for a neutral polymer slightly decreases the packing time. As pointed out in Ref. [8], the entropic penalty to pack a polymer is large. Based on thermodynamic grounds, an increase in temperature would make this penalty even more due to larger configurational entropy outside the capsid. Hence, one would expect that packing to be more difficult at higher temperatures. However, higher temperatures also help the polymer inside the capsid to ralax more rapidly making the packing faster [20]. The effect seen in our simulations is small due to hydrodynamic interactions, becoming larger when these interactions are turned off (see Fig. 3). This strengthens the argument that polymer relaxation inside the capsid is the driving force towards faster packing. (Hydrodynamic interactions can be destroyed in our simulations by assigning to each solvent particle a random velocity generated from a normal distribution of variance 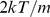 after each collision step, where 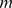 is the mass of a solvent particle.)

The effect of temeperature is noticeably reversed when the polymer is charged (Fig. 2b). We observe that packing is reduced appreciably at higher 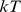, to the point that we do not achieve full packing when 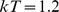. Here, the electrostatic forces become larger at higher 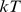 (see Eq. (4)), thereby, hampering packing once enough beads are packed inside.

**Figure 4:**
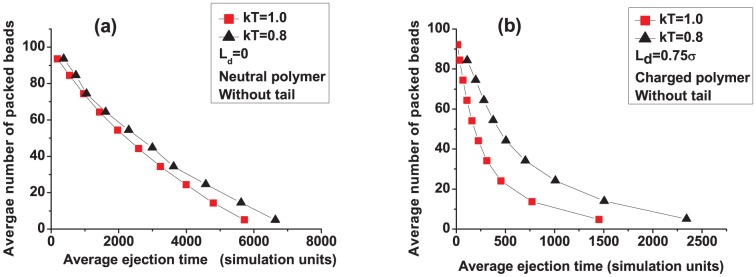
Effect of temperature on ejection of a semiflexible polymer: (a) polymer is neutral and (b) polymer is charged ( 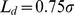
 **).**

Ejection shows the opposite trend with temperature. Fig. 4 shows the number of beads remaining in the capsid during ejection vs. time at 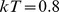 and 1.0. Regardless of whether the polymer is neutral or charged, ejection is slower at lower temperatures. We interpret our results by considering the entropy of the system. A lower temperature induces the polymer to adopt fewer configurations outside the capsid, resulting in a lower entropic gain which slows the ejection process. For the charged polymer, there is the additional factor that electrostatic forces in our simulations are reduced at lower temperatures (Eq. (4)) which result in a lower ejection force from the capsid. We note that, as surmised in experiments [5], [6], the closing of the pore will also make a difference. However, here we study the thermodynamic effects of 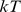 without assuming any conformational changes in the pore. It is interesting that there is an effect also in this limit.

### Effect of Capsid Tail

**Figure 5:**
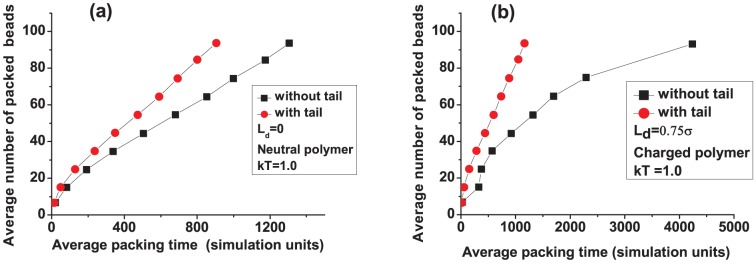
Effect of the capsid tail on packing of a semiflexible polymer: (a) polymer is neutral and (b) polymer is charged ( 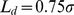
 **).**

**Figure 6:**
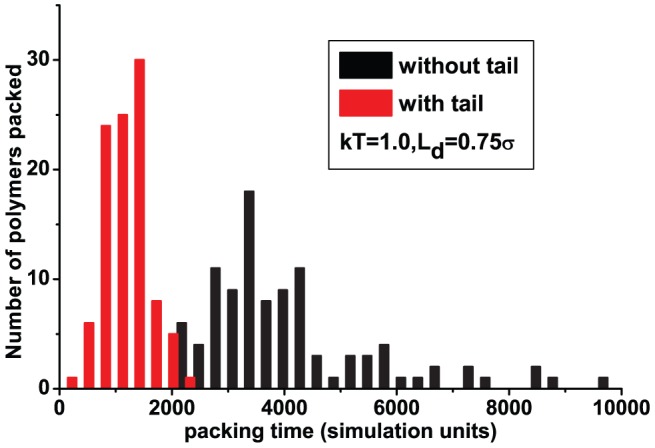
Packing time distribution of charged (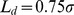) semiflexible polymers.

Figs. 5a and 5b show the effect of the capsid tail on packing of neutral (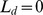) and charged (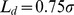) polymers, respectively. It is easier on average to pack both polymers into a capsid with a tail. The entropic reduction due to the tail makes packing a neutral polymer more efficient. However, the speedup for the charged polymer is surprisingly large (a factor of 3 to 4). We further plot in Fig. 6 the packing time distribution for the charged polymer. The distribution is rather wide for a tailless capsid but narrow and peaked at shorter times when the tail is included.

**Figure 7:**
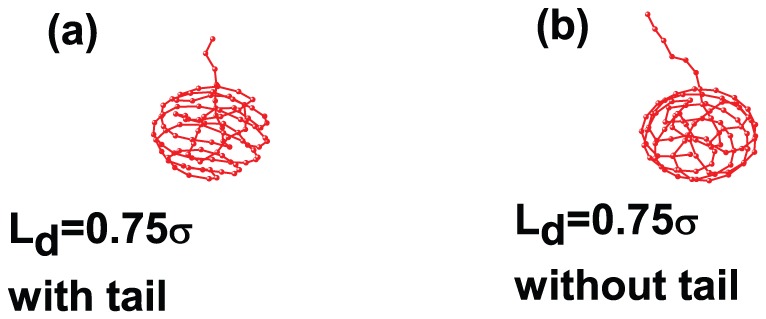
The structure of a charged(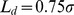), semiflexible polymer inside the capsid at 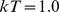: (a) with the tail and (b) without the tail.

We explain such state of affairs by considering the arrangement of the polymer inside the capsid. Fig. 7a (7b) gives the polymer structure inside a capsid with (without) a tail. When the tail is present, the arrangement is more ordered, which lowers the packing free energy and, as a result, drives packing to larger speeds. At 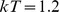, we obtain a greater packing degree for the charged polymer compared to the tailless capsid (Fig. 2b), but still not full. Evidently, the electrostatic forces are still large enough to prevent full packing. (We note that we do not find much effect of the temperature on the polymer configuration inside the capsid. The reason is that, within the temperature range used in this study, electrostatic forces in our simulations are still large and do not allow substantial structural changes.)

By including torsional effects, some recent Monte Carlo simulation studies [21] have achieved the experimentally observed inverse-spool polymer structure inside the capsid. On the other hand, crystal structure studies [18] suggest that DNA may twist while being packed in the 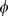
29 phage. Based on this, Spakowitz and Wang [22] have run Brownian dynamics simulations including torsional effects, where one end of the polymer is attached to the capsid wall and, in addition, the polymer is twisted while being fed into the capsid. A spool sturcture has also resulted. These studies suggest that torsional properties may be of importance in determining the final polymer configuration when packed inside a capsid. However, recent simulations of packing into P4 phage by Rollins et. al. [11] have shown that including torsional effects change the free energy of packing by at most 10%, which is rather small. As the study points out, this is mainly because the free energy depends on polymer entropy loss during packing and on electrostatic repulsions. This means that, at least at a qualitative level, the packing and ejection dynamics are not much affected by torsional effects. In the current paper, we are able with the use of a simple model to reproduce the general features of the experimental results (see Refs. [1], [2] and below) regardless of the exact polymer structure inside the capsid.

**Figure 8:**
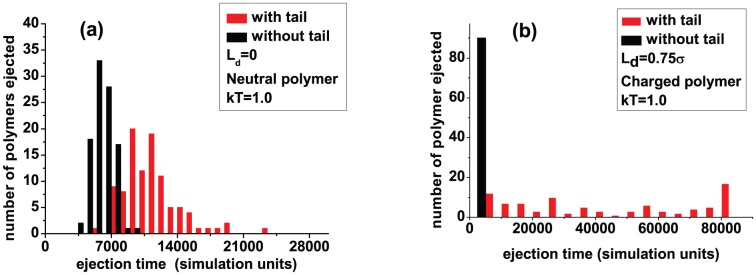
Effect of the tail on ejection of a semiflexible polymers: (a) polymers are neutral and (b) polymers are charged (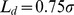).

In contrast to packing, ejection is harder for both neutral and charged polymers with a tail. Fig. 8(a) shows the ejection time distribution for neutral polymers in a good solvent. The mean ejection time in our simulations for a capsid with a tail is practically double that of a tailless capsid, with a wider distribution.

In a good solvent the polymer inside the capsid has larger entropy than when in the tail. Therefore, this opposing entopic force against the ejection slows the ejection process.

The ejection time distribution with a tail for charged polymers (Fig. 8b) shows a different behavior. It shows a sharp peak at 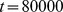 simulation units (corresponding to 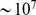 MD steps), the maximum time allowed for a simulation run. The rest of the distribution is flat at earlier times when compared to that ensuing from a tailless capsid. Clearly many polymers failed to eject during the simulation run, presumably needing more time for full ejection. This is surprising, considering the fact that the presence of electrostatic forces should induce complete ejection in reasonable time even when the tail is included.

**Figure 9:**
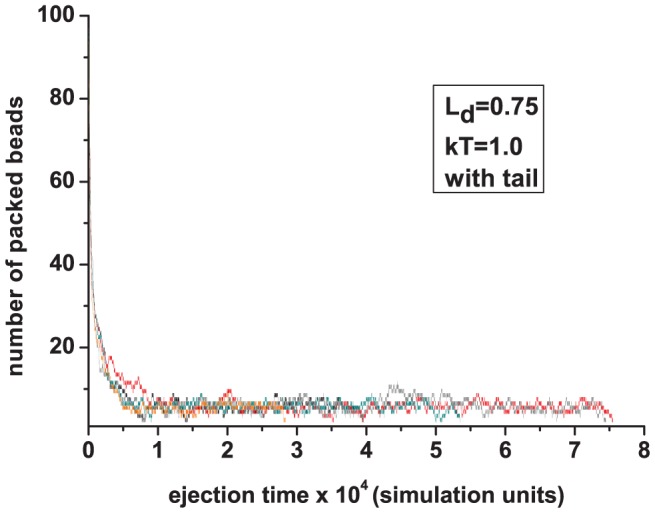
Beads remaining in a capsid with tail as a function of time for 5 individual charged polymers (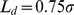).

To better understand this result, we plot in Fig. 9 the number of beads remaining in the capsid with a tail as a function of ejection time for some individual polymers. The large tail found at later times suggest that the last few beads are trapped in the tail, which is similar to what is found experimentally with ejection of 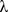 DNA in 10 mM NaCl buffer [3].

The slowdown in ejection can be explained based on entropic arguments. The polymer entropy (whether the polymer is charged or not) inside the capsid is larger than its entropy when in the tail. This offers opposing entopic force against the ejection. For the charged polymer there is the additional effect that the last few beads are trapped for a long time in the tail. This reflects the fact that even though most of the polymer is out, its entropy when outside the capsid is greatly reduced due to electrostatic interactions (the polymer adopts more extended configurations outside), which makes it difficult to pull the last few beads out.

**Figure 10:**
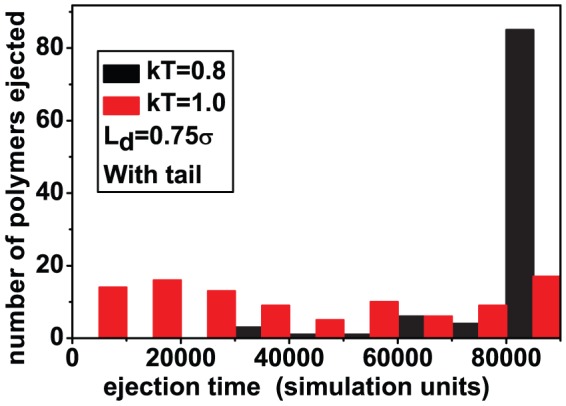
Effect of temperature on the ejection of charged (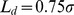) polymers from a capsid with tail.

Fig. 10 shows the effect of temperature on the ejection with the tail for charged polymer. It can be seen that as temperature decreases the ejection becomes much slower. The slowdown is markedly greater compared to the tailless capsid case (see Fig. 4b). It is not only entropic reduction in the tail that plays a role here, but also the smaller ejection force (Eq. (4)), in addition to better bead trapping mentioned above.

## Conclusion

In summary, we studied the effect of temperature and capsid tail on the packing and ejection processes of neutral and charged semiflexible polymers. We find that for a high packing force and at higher temperatures the packing time decreases for the neutral polymer due to larger thermal fluctuations that aid in feeding the beads into the capsid, whereas packing is harder for a charged polymer because of the increase in electrostatic forces.

On the other hand, decreasing the temperature slows the ejection process for both neutral and charged polymers; for the former it is because the entropic gain upon ejection is reduced, while for the latter it is the reduction in electrostatic forces that has a dominant role.

We find that including the capsid tail facilitates easier packing but ejection becomes slower. These effects are much larger for charged polymers. It is interesting that the tail plays two opposite roles: it can influence the polymer configuration inside the capsid to a degree that packing more charged beads becomes feasible at higher temperatures, despite the larger repulsive electrostatic forces; on the other hand, it acts as a trap during ejection, further delaying the polymer in freeing itself completely from the capsid. Our results suggest that both entropic and electrostatic forces are critical to the polymer behavior.

It would be interesting to include friction and study its role on ejection, which could be of importance [3]. Further, simulations could provide a way through which one can gain some insight into ejection *in vivo*, which is currently not well understood. This is left for future work.
